# The impact of preoperative anxiety on patients undergoing brain surgery: a systematic review

**DOI:** 10.1007/s10143-021-01498-1

**Published:** 2021-02-19

**Authors:** Vittorio Oteri, Anna Martinelli, Elisa Crivellaro, Francesca Gigli

**Affiliations:** 1 Department of General Surgery and Medical Specialties, University of Catania, Catania, Italy; 2 University of Padova, Padova, Italy; 3 University of Milano, Milano, Italy

**Keywords:** Neurosurgery, Brain surgery, Preoperative anxiety, Perioperative care, Quality of life, Systematic review

## Abstract

Preoperative anxiety is a common reaction exhibited by up to 80% of patients who are scheduled for surgical procedures and characterized by psychological and physical changes which may affect their perioperative period. Our aim is to report the most up-to-date evidence on preoperative anxiety in brain surgery patients through a systematic analysis of the studies produced in the last decades. We performed a systematic review of literature by searching PubMed, Embase, and Cochrane Library databases. Data were extracted using the Population, Intervention, Comparison, Outcome framework and critically analyzed. PRISMA guidelines were applied, and the risk of bias of the included studies was assessed using the Risk of bias (RoB) 2 and ROBINS tools, as was the methodological quality, following GRADE criteria. We included 27 articles, accounting for 2558 patients in twelve different countries. The prevalence of preoperative anxiety ranged from 17 up to 89%, higher in female patients. Preoperative anxiety was associated with lower quality of life and cognitive performance, higher need for information, poorer memory and attention, longer hospitalization, depressive symptoms, and increase of physical disability; no correlation with survival rate was found. Seven randomized controlled trials attested the efficacy of acupuncture, music therapy, virtual reality, and pharmacological support in lowering anxiety levels. Preoperative anxiety is a common phenomenon that could negatively affect the perioperative period of brain surgery patients: this is something that should not be neglected to achieve better care through early prevention and optimal management.

**Supplementary Information:**

The online version contains supplementary material available at 10.1007/s10143-021-01498-1.

## Introduction

Preoperative anxiety is a common finding in patients who are scheduled for surgical procedures [1, 43, 53]. It is estimated that among patients admitted for surgery 25 to 80% of them experience preoperative anxiety [43]. The anxiety severity widely differs among patients and it is usually associated with the following: sociodemographic factors, such as sex, age, and educational background; psychosocial factors, like for example baseline anxiety levels or psychiatric comorbidities, personality traits, social support, and coping style; factors regarding the specific pathology for which surgery is required, possible complications of the operation, method of anaesthesia, and preoperative information [5]. In particular, surgery and general anaesthesia still represent two of the most traumatic events in the patient’s life [13], characterized by three distinct negative aspects: the fear of the unknown, the idea of being sick, and the possibility of life-ending [9].

Emerging pieces of evidence show how preoperative anxiety starts as soon as the procedure is planned and reaches its peak on the day of surgery [27]; this is further confirmed by associated physical changes [10, 33], such as increased hormones and acute phase’s protein release, tachycardia episodes, hypertension, rise in body temperature [33], fluid and electrolyte imbalances, diminished immune responses, and longer wound healing [10], which can affect the surgical outcome and the postoperative recovery [34] and lead to the increase of the dosage of anaesthetics and sedatives given on the day of surgery [30, 43] with a consequent higher risk of adverse events and interactions. Likewise, patients with anxiety tend to have a longer hospital stay, a decreased postoperative satisfaction, and to be less compliant with rehabilitation and occupational therapy [41].

During the last decade, a relevant number of studies were focused on preoperative anxiety in patients undergoing neurosurgical procedures, analyzing possible causes, potential predictors, influence on surgical outcomes, and strategies to decrease the occurrence of anxiety. However, there are no records of a systematic review of the effects of preoperative anxiety on brain surgery patients. We hypothesize that preoperative anxiety has a significant impact on patients’ well-being, subjective experience, and surgical outcomes, especially on those undergoing brain surgery, where procedures can be extremely complex and often performed in awake conditions [35]. The aim of our systematic review is to report, organize, and critically analyze, both in a quantitative and qualitative way, the most up-to-date evidence on the preoperative anxiety phenomenon in patients undergoing brain surgery.

## Materials and methods

### Literature search strategy

We conducted this review following the Preferred Reporting Items for Systematic Reviews and Meta-Analyses (PRISMA) guidelines [28] and methodological advices from the Cochrane Handbook for Systematic Reviews of Interventions [22].

A comprehensive search was performed on three medical electronic databases (PubMed, Embase, and Cochrane Library) up to the 13th of February 2020. Our main aims were (1) to investigate the characteristics of preoperative anxiety phenomenon among brain surgery patients, (2) to identify its impact on their perioperative period, and (3) to evaluate methods to assess it and interventions to lower it. To achieve the maximum sensitivity of the search strategy, we combined these terms: ((Anterior Temporal Lobectomy) OR (Brain Tissue Transplantation) OR (Cerebral Decortication) OR Hemispherectomy OR (Cerebrospinal Fluid Shunts) OR (Ventriculoperitoneal Shunt) OR Ventriculostomy OR Craniotomy OR (Decompressive Craniectomy) OR Trephining OR Axotomy OR Hypophysectomy OR (Microvascular Decompression Surgery) OR Neuroendoscopy OR Pallidotomy OR Psychosurgery OR (Split-Brain Procedure) OR (Stereotaxic Techniques) OR Neuronavigation OR Radiosurgery OR neurosurgery OR Neurosurgeries OR neurosurgical OR (Neurosurgical Procedure) OR brain OR Encephalon OR (brain surgery)) AND (anxiety OR (anxiety disorder) OR Nervousness OR Hypervigilance) AND (preoperative OR pre-operative OR (Preoperative Period) OR (Preoperative Care)) as either keywords or MeSH terms. The reference lists of all included articles, previous literature reviews on the topic, and top hits from Google Scholar were reviewed for further identification of potentially relevant studies, to ensure completeness of this review. To avoid overlapping with other ongoing reviews, we first searched on PROSPERO website for any similar review and then registered our review protocol (ID CRD42020177142).

### Selection criteria

Eligible studies for our systematic review included those investigating every aspect of the phenomenon of preoperative anxiety on patients involved in brain surgery procedures and reporting all types of outcomes. Studies focused exclusively on spine surgery were our exclusion criteria.

**Fig. 1:**
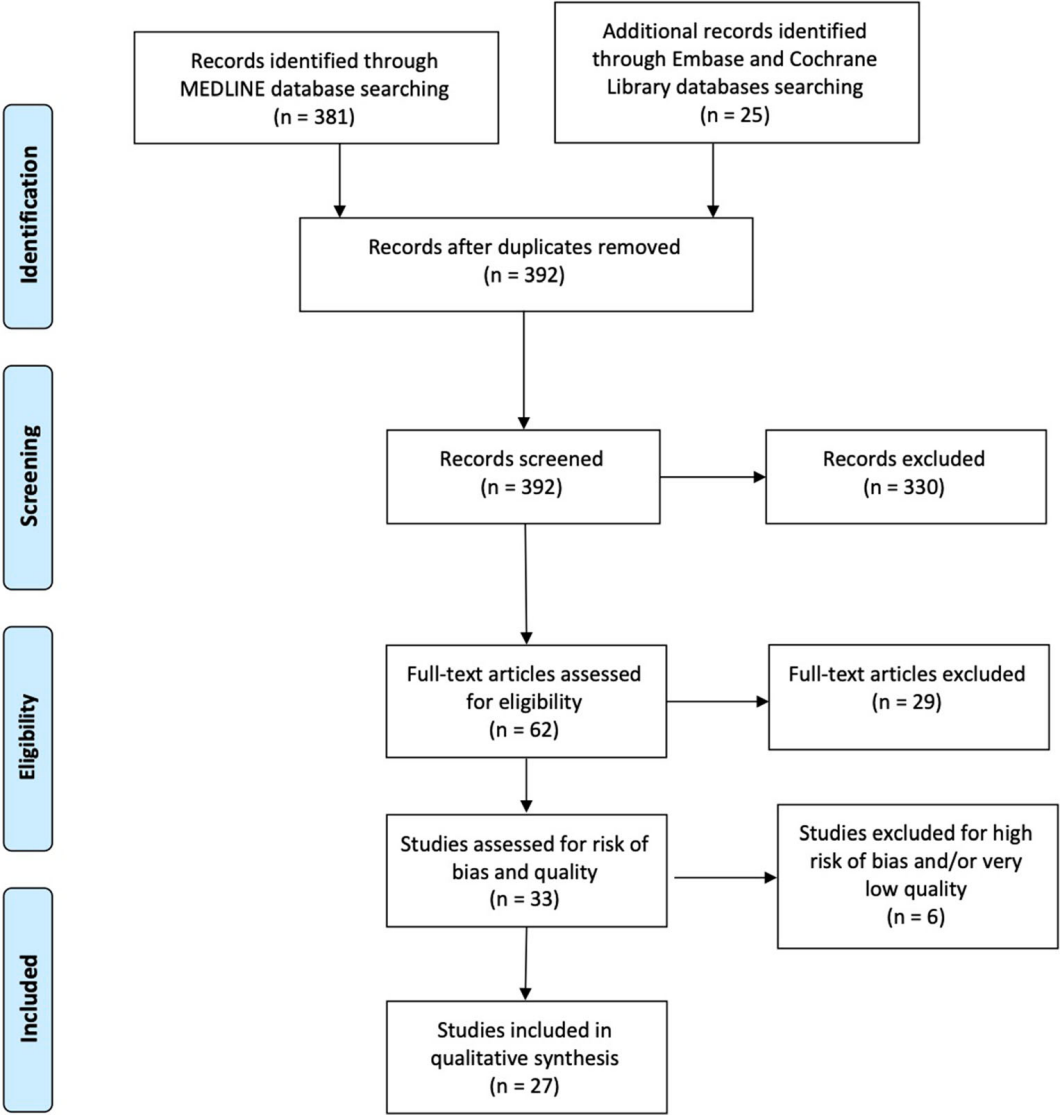
PRISMA flow diagram of study selection

Primary screening of the titles and abstracts was performed by adding studies of any level of evidence published in peer-reviewed journals written in English. Additionally, we excluded studies in which data were not accessible, missing, without an available full text, or not well reported. Duplicates, abstracts, case reports, conference presentations, reviews without original data, editorials, and expert opinions were excluded. Two authors (A.M. and E.C.) performed the search and evaluated the articles independently. An experienced researcher in systematic reviews (V.O.) solved cases of doubt. At the beginning of the procedure, each investigator read the abstracts of all the articles, selected the relevant ones according to both inclusion and exclusion criteria, and then compared the results with the other investigators, with err on the side of inclusion. After 4 weeks, the same studies were reread to establish the agreement of the investigators about articles’ selection. No disagreement was observed among the investigators. Figure 1 depicts the PRISMA flow diagram of study selection.

### Data extraction and criteria appraisal

All data were extracted from articles’ text, tables, and figures, using the Population, Intervention, Comparison, Outcome (PICO) framework [39] and included title, year of publication, study design, sample size, study population, patients’ characteristics, intervention and comparator (where applicable), outcomes, and conclusions. One investigator extracted the data from the full-text articles to Excel spreadsheet structured tables to analyze each study in a descriptive fashion. The other investigator independently double-checked the extraction of primary data from all the articles. Two investigators independently reviewed each article (A.M. and E.C.). Discrepancies between the two reviewers were resolved by discussion and consensus. The final results were reviewed by the senior investigator (V.O.).

### Risk of bias assessment

Risk of bias (RoB) assessment in full text of all the studies selected was performed according to the following: the ROBINS-I tool [44] for non-randomized trials, which evaluates seven domains of bias to reach for an overall RoB judgment (low, moderate, serious, critical); the RoB 2 tool [45] for randomized trials, which evaluates five domains of bias to reach for an overall RoB judgment (low, some concerns, high).

Two authors performed the assessment (F.G. and V.O.) independently. Inter-rater agreement was 92%. Any discrepancy was discussed and solved. Tables 1aS and 1bS in Online Resource outline the RoB assessment. We excluded articles with serious RoB.

### Study quality assessment

The research methodology quality assessment was completed applying the (GRADE) approach [40]. The critical determinant of the starting grade of a study is its design: randomized trials provide high-quality evidence, whereas observational studies provide low-quality evidence; the assessment continues with subsequent evaluation of five factors that can rate down the quality of evidence and three factors that can rate it up. This leads to an overall evaluation of a body of evidence in one of four grades (high, moderate, low, very low).

The assessment was performed by two authors (F.G. and V.O.) independently. Inter-rater agreement was 94%. Any discrepancy was discussed and solved. Table 2S in Online Resource outlines the quality assessment.

We excluded articles evaluated as very low quality.

## Results

### Study characteristics

We identified 27 studies which evaluated the impact of preoperative anxiety in patients undergoing brain surgery. They account for a total of 2558 participants (45% males, 55% females), with a mean age of 49 years. These studies were conducted in the wards of anesthesiology, neurology, and neurosurgery of twelve different countries: twenty-one studies (78%) in Europe, four (15%) in America, and two (7%) in Asia.

Thirteen studies referred to resection of brain tumors [6–8, 12, 14–16, 26, 29, 31, 32, 47, 50], two studies did not specify the type of neurosurgery considered [23, 52], six studies were focused on craniotomy [3, 4, 17, 30, 42, 55], and four studies on awake craniotomy [21, 35, 37, 38]; the remaining studies included patients undergoing seizure surgery [20, 54].

Among the studies related to brain tumor surgery, the most represented type of tumor was meningioma (6/13 studies, 46%) [6, 7, 12, 16, 31, 47], followed by glioma (4/13 studies, 31%) [6, 7, 12, 31], acoustic neuroma (3/13 studies, 23%) [6, 12, 31], and pituitary adenoma (2/13 studies, 15%) [6, 12].

Seven studies were randomized controlled trials [3, 4, 23, 42, 50, 52, 55], thirteen were prospective studies [6, 7, 12, 15, 16, 20, 21, 26, 32, 37, 38, 47, 54], and seven were cross-sectional studies [8, 14, 17, 29–31, 35].

Twelve studies used the Hospital Anxiety and Depression Scale (HADS), which is designed to measure the severity of anxiety psychological symptoms especially in people with somatic complaints, as the assessment method of preoperative anxiety [6–8, 14–17, 21, 29, 31, 32, 35]. Six studies utilized the Amsterdam Preoperative Anxiety and Information Scale (APAIS) [4, 14, 15, 17, 30, 52], which relies on psychological symptoms and is recommended for discriminating between anxiety from surgery or anesthesia and for assessing information requirement of patients. Six studies used the State-Trait Anxiety Inventory (STAI) [12, 23, 37, 38, 47, 52], a psychological measure that assesses someone’s state and trait anxiety. Six studies utilized a Visual Analog Scale (VAS) [4, 17, 23, 30, 50, 55]. Other scales used only once are listed below. The Post-Traumatic Stress Scale (PTSS) [47] was used to assess physical and psychological symptoms of post-traumatic stress disorder. The Anxiety Sensitivity Index (ASI) [47] was utilized to investigate physical, social, and cognitive traits of anxiety. The State-Trait Operation Anxiety (STOA) [17] is a psychological scale that was used to discriminate between state and trait anxiety. The Acute Stress Disorder Scale (ASDS) [15], which relies on psychological items, was utilized to diagnose acute stress disorder and to predict post-traumatic stress disorder. The Pain Anxiety Symptoms Scale (PASS) [37], used to detect fear of pain, is a specific assessment method which works with cognitive, behavioral, and physiological domains. The CCEI (Crown-Crisp Experiential Index) [26] was utilized because it represents a measure of psychological and physical symptoms of psychoneurotic pathology and anxiety. Lastly, also a Numerical Rating Scale (NRS) [42] was used.

Eighteen studies used only one scale [3, 6–8, 12, 16, 20, 21, 26, 29, 31, 32, 35, 38, 42, 50, 54, 55] to measure anxiety, six used two scales [4, 14, 23, 30, 37, 52], two studies used three scales [15, 47], and one used four scales [17] in the same cohort of patients.

The most common time point of the preoperative assessment was the day before surgery [17, 23, 29, 30, 55]; other frequent ones were on surgery day [4, 21, 23, 50] and within 1 [15, 16, 26, 31] or 2 [30, 35] weeks prior to surgery. Furthermore, six studies included a follow-up during postoperative period, up to 1 month [12, 54], 3 months [12, 21, 26, 47, 54], 6 months [12, 16], and 1 year [12, 26, 47] after surgery.

A summary of the study characteristics is available in Table 3S in Online Resource.

The findings reported in the following paragraphs are summarized in Table 1.

**Table 1:** Summary of results

Topic	Main findings
Characteristics of preoperative anxiety and factors correlated	Prevalence of clinically relevant (mild or moderate at least) preoperative anxiety ranged from 17 to 89% among patients admitted for neurosurgery, with severe/high anxiety affecting up to 55% of patients; preoperative anxiety was higher in women than men, and it was mainly related to surgery outcome and anesthesia. The majority of the articles found no significant correlation between preoperative anxiety and histological characteristics, side location, and occurrence or recurrence of brain neoplasm. Previous trauma and negative experience, lack of perceived social support, lower preoperative free T3 concentration, and high trait anxiety were also associated with higher preoperative anxiety.
Preoperative anxiety and preoperative period	Preoperative anxiety could lead to a lower health-related quality of life, lower cognitive performance, and self-perception of worse memory and attention during the preoperative period, worsening the perception of the patient’s own capability and safety during surgery and anesthesia.
Preoperative anxiety and postoperative period	Preoperative anxiety could have implications on various aspects of the postoperative period of brain tumor patients, such as depressive symptoms, decrease in quality of life, and increase of physical disability, although no correlation between preoperative anxiety and survival rate was found; regarding seizure surgery patients, the findings are conflicting.
Awake surgery	Anxiety before awake surgery does not seem to differ much from anxiety occurring before traditional brain surgery; no associations between preoperative anxiety and intraoperative tests were noted. Postoperative pain and psychological status could be affected by preoperative psychological symptoms.
Interventions to lower preoperative anxiety	Based on seven RCTs, several methods to reduce preoperative anxiety proved to be effective: both pharmacological, such as pregabalin, flupirtine, and oxazepam, and non-pharmacological, such as acupuncture, virtual reality, and music therapy; skin surface warming had no influence on anxiety in this group of patients.

### Characteristics of preoperative anxiety and factors correlated

From the analysis of eighteen studies, also considering their quality and RoB, it was found that the prevalence of clinically relevant (mild or moderate at least) preoperative anxiety ranged from 17% [6] to 89% [30] among patients admitted for neurosurgery [7, 8, 12, 15–17, 31, 32, 47, 52, 54], with severe/high anxiety affecting up to 55% of patients [30]; preoperative anxiety was higher in women than men [12, 14, 15, 17, 30, 32], and it was mainly related to surgery outcome and anesthesia [14, 30, 55]. The majority of the articles found no significant correlation between preoperative anxiety and histological characteristics, side location, and occurrence or recurrence of brain neoplasm [7, 12, 16, 26, 29, 32, 54]. Previous trauma [16] and negative experience [17], lack of perceived social support [16], lower preoperative free T3 concentration [6], and high trait anxiety [17] were also associated with higher preoperative anxiety.

Considering the correlation between sex and preoperative anxiety, Perks et al. [30] referred that, in patients admitted for craniotomy or transsphenoidal surgery, preoperative anxiety was significantly higher in females compared with males (VAS, 5.8 ± 2.8 vs 4.6 ± 2.5, *P* < .05) and female sex was identified as the only risk factor which led to higher levels of anxiety (*P* < .02). This study was evaluated as low quality and low RoB.

Only Mainio et al. [26] found out a significant difference in preoperative anxiety between patients with right and left hemisphere tumor (5.75 ± 3.32 vs 3.59 ± 3.12, *Z* =−2.14, *P* = .032). This article was evaluated as low RoB and low quality.

Pringle et al. [32] referred that patients with a meningioma obtained higher scores of anxiety compared with patients with other types of brain tumor. This study was evaluated as unclear RoB and low quality.

### Preoperative anxiety and preoperative period

Five studies [8, 14, 15, 30, 31], also considering their quality and RoB, tended to demonstrate that preoperative anxiety could lead to a lower health-related quality of life [8], lower cognitive performance [15], and self-perception of worse memory and attention during the preoperative period [31], worsening the perception of patient’s own capability and safety during surgery and anesthesia [14, 30].

For the 172 tumor patients studied by Goebel et al. [15], high APAIS score was negatively correlated (*P* < .05) with visuospatial performance (VOSP number location, *r* =−.188), flexibility (FPT, *r* =−.214), and strategic performance (FPT, *r* =−.202). After accounting for age and education, the APAIS score was not associated with neuropsychological test results (*P* > .2). Comparison of extreme groups according to APAIS score showed that higher anxiety correlated with worse performance in verbal working memory based on the Digit Span Backwards (medium group difference of DSB, *r* = .99 ± .39) (*P* = .013). This study was evaluated as low RoB and low quality.

Bunevicius et al. [8] found that in 200 brain tumor patients, preoperative anxiety (HADS-A) was associated with lower scores on all aspects of health-related quality of life (HRQoL): physical functioning (*β* =−0.29), role limitations due to physical (*β* =−0.30) and emotional problems (*β* =−0.32), fatigue (*β*=−0.39), emotional well-being (*β* =−0.47), social functioning (*β* = 0.20), pain (*β* =−0.25), and general health (*β* =−0.49) (all *P* < .001). Considering multivariable linear-regression analyses, HADS-A score was the strongest independent predictor of role limitations due to physical problems and emotional problems (both *β* =−0.28, *P* < .001), as well as general health (*β* =−0.27, *P* < .001). This study was evaluated as moderate RoB and low quality.

### Preoperative anxiety and postoperative period

The results of five studies [7, 12, 20, 47, 54] may suggest, considering their quality and RoB, that preoperative anxiety could have implications on various aspects of postoperative period of brain tumor patients, such as depressive symptoms [12], decrease in quality of life (QOL), and increase of physical disability [47], although no correlation between preoperative anxiety and survival rate was found [7]; regarding seizure surgery patients, the findings are conflicting [20, 54].

D’Angelo et al. [12] found that, in 114 patients who underwent brain tumor surgery, the presence of trait anxiety at the enrollment was correlated with current depression at 1 month after surgery (*P* = .0009) and at 3 months after surgery (*P* = .003); the logistic regression analysis confirmed that preoperative trait anxiety was the main determinant of depression at 1-month follow-up.

Guarnieri et al. [20] discovered that, in 186 patients treated for MTLE-HS, 10 (5.4%) of them manifested anxiety disorder (AD) according to DSM-IV criteria, and there was an independent association between preoperative AD and the presence of neurological symptoms after anterior temporal lobectomy (adjusted HR = 2.48, 95% CI = 1.25–4.93, *P* = .009). Particularly, subcategory E1B (Engel’s class >= E1B, free of disabling seizures, with auras only) was strongly associated with preoperative AD (HR = 3.50, 95% CI = 1.31–9.31, *P* = .012). These two studies were evaluated as moderate quality and moderate RoB.

Wrench et al. [54] considered 60 seizure surgery patients, among whom 43 underwent temporal and 17 extra-temporal resections, and found no relation between patients predicted to develop the burden of normality and preoperative anxiety (phi =−.185; *P* > .05). This study was assessed as low quality and low RoB.

### Awake surgery

Analyzing four studies [21, 35, 37, 38], also considering their quality and RoB, anxiety before awake surgery does not seem to differ much from anxiety occurring before traditional brain surgery [35, 38]; no associations between preoperative anxiety and intraoperative tests were noted [38]. Postoperative pain and psychological status [21] could be affected by preoperative psychological symptoms and especially pain-related anxiety [37] must not be underestimated.

In the study carried out by Hejerati et al. [21] preoperative anxiety was associated both with anxiety levels 3 days after surgery (*ρ* = 0.80, *P* < .001), and 3 months after surgery (*ρ* = 0.68, *P* < .01). Preoperative anxiety also correlated with pain intensity the day before surgery (*ρ* = 0.45, *P* < .05) and with pain (*ρ* = 0.49, *P* < .05), pain intensity (*ρ*=0.52, *P* < .05), and pain interference with daily activities (*ρ* = 0.72, *P* < .05) 3 days after surgery.

Santini et al. [37] found that, in the preoperative period, cognitive anxiety (PASS-CA) was positively correlated with depression (BDI, *ρ* = 0.515, *P* < .05), and that preoperative pain-related anxiety (PASS) total scores were correlated with pain felt during and at the end of the operation (respectively *ρ* =−1.00, *P* < .05 and *ρ* = 0.866, *P* < .05). These two studies were classified as moderate RoB and low quality.

### Interventions to lower preoperative anxiety

Considering the findings reported by seven RCTs [3, 4, 23, 42, 50, 52, 55], also in relation to their quality and RoB, it emerges that several methods could be effectively applied to lower preoperative anxiety in patients awaiting brain surgery, both pharmacological, such as pregabalin [42], flupirtine [55], and oxazepam [3], and non-pharmacological, such as acupuncture [52], virtual reality [4], and music therapy [50]; skin surface warming had no influence on anxiety in this group of patients [23].

In particular, Shimony et al. [42] selected two groups of 50 patients each, receiving 150-mg pregabalin capsules or 500-mg starch capsules (placebo) the night before surgery and 1.5 h before surgery: both groups had similarly high levels of self-rated anxiety at the time of the recruitment (NRS [0–10], pregabalin vs placebo, mean 4.92 ± 2.95, CON 5.62 ± 2.71, *P* = .28), but the anxiety level prior to surgery appeared to be significantly lower in the pregabalin group than in the control one (NRS [0–10], pregabalin vs placebo, mean 3.13 ± 2.3, CON 4.25 ± 2.65, *P* = .04); additionally, only the pregabalin group experienced a significant reduction in anxiety levels from admission to prior surgery (*P* = .04). This study was assessed as high quality and low RoB.

On the non-pharmacological side, Wiles et al. [52] reported that 62 patients, who received acupuncture at the EX-HN3 (Yintang) point before surgery, showed a statistically significant reduction in anxiety level after 30 min (STAI-S6 and APAIS, preintervention vs post-acupuncture, median [IQR], 46.7 [36.7–53.3] vs 40.0 [30.0–46.7], *P* < .001 and 10 [6–13] vs 7 [4–10], *P* < .001). No change was seen in the 62 patients of the control group who received no intervention. This study was evaluated as high quality and some concerns RoB.

## Discussion

The findings of this systematic review depict preoperative endorsement of anxiety symptoms in neurosurgery as a common phenomenon capable of deteriorating patients’ cognitive functioning, QOL, and general well-being. Preoperative anxiety should be assessed due to the possible negative influence on various parts of the perioperative period, and assessment tools and interventions are needed in order to detect and treat preoperative anxiety effectively; in this regard, the consultation with specialty mental health experts really takes on value to follow integrated health and best practices: rehabilitation psychologists, neuropsychologists, or health psychologists (practicing psycho-oncology) should be included permanently in the team of professionals caring for neurosurgical patients, assuring the best assessment and management of neuropsychological and emotional concerns of the patients during their pathway of care.

Several interventions, both pharmacological and non-pharmacological, displayed some efficacy in lowering preoperative anxiety levels in neurosurgical patients; nevertheless, these promising results have to be taken with caution and will need further assessment, given that they rely on individual studies with a limited number of participants, not meeting the class 1A evidence for recommendation.

The pharmacological interventions, which seemed to prove effective in reducing anxiety without major adverse events, could be a favorable solution in order to avoid side effects of common anxiolytic used such as benzodiazepines, barbiturates, and opioids, especially in a neurosurgical setting, where prompt and sustained regaining of consciousness is desired for an early neurological assessment. Pregabalin, a gabapentinoid that acts as co-adjuvant opioid, also improves sleep quality, lowers postoperative pain, nausea, and vomiting, and has an opioid-sparing effect; flupirtine, a centrally acting non-opioid analgesic, is very useful at minimum analgesic dose, also in conjunction with behavioral interventions, and has good properties like preservation of respiratory functions, preemptive analgesic effects, and antiepileptic properties; fast-dissolving oxazepam formula is highly effective and short-acting, with minimal side effects and high acceptability.

On the other side, non-pharmacological interventions could be useful, alone or in association with drugs, to lower preoperative anxiety and, if these results will be corroborated by future studies, they should be integrated into the standard of care of these patients. Acupuncture could achieve up to 30% reduction of anxiety and it is easy to administer also by a non-specialist practitioner (such as medical students) and in a busy preoperative environment [2]. VR is a novel intervention allowing the immersion of patients into the perioperative setting [19], increasing their familiarity with it by aligning their expectations to reality. This was proved to decrease overall anxiety, solve uncertainties, increase preparedness, strengthen the physician-patient relationship, and improve patient satisfaction. Music intervention could also provide a better perception of hospitalization and more relaxation while encouraging families to participate actively, which could benefit as well in reduction of anxiety during waiting periods. Other behavioral interventions such as counseling, distraction, attention focusing, and relaxation procedures could be effective too and should be investigated, bearing in mind that these and other kinds of behavioral and psychotherapeutic interventions might suffer some limitations by patients with intellectual disability, low health literacy, or poor education and reading ability.

Preoperative levels of anxiety were correlated with worsening in postoperative depression [12], QOL, and physical disability [47], demanding a strict follow-up for anxious brain tumor patients; although its association with survival remains questionable, preoperative anxiety could represent an important novel prognostic factor to complement the established ones in evaluating the postoperative course of patients.

The information and the reassurances that patients receive through the preoperative doctor-patient communication is demonstrated to decrease anxiety [24, 49]; nevertheless, it is clear that this usual care may not be enough for some individuals: indeed, medium to high levels of preoperative anxiety were found in patients that had already been assessed and instructed about the procedure by anaesthetists and surgeons.

In an awake surgery setting, anxiety plays an important role considering that the attitude and collaboration of the patients are crucial for the procedure and its planning, and it could also lead to unsuitability for surgery [48]. Moreover, although routine tests are capable to screen patients and avoid major complications, more sensitive tests, tools, and criteria are needed to detect warning signs [11] in anxious patients that could have poor compliance and need for longer hospitalization [21].

### Implications and possible practical applications

Symptoms of depression and anxiety often mimic those commonly found in patients with brain tumors (that is, tiredness and fatigue), and sometimes, the neurosurgeon focuses his or her attention only on the organic features [51]; it also emerged that subjective affective conditions were not associated with objective medical data, stressing that the impact on patients well-being is predictable only if specifically assessed [15].

For these reasons, the psychometric evaluation gains great importance in the global evaluation of neurosurgical patients, and especially, but not only, for those affected by brain tumors. The health care team should integrate the daily management of patients with instruments assessing anxiety, be used along the process of care, from admission to the days after surgery and beyond.

### Future directions of research

Whereas for some establishments the evidence collected is strong and enough in number, such as for the association of anxiety with female sex, other specific findings should be taken with caution and would need more and larger studies, preferably conducted in homogenous and comparable ways, to be confirmed, because their strength relies on too little cases.

Several perioperative factors have been negatively correlated with preoperative anxiety; although it is intuitive that these factors can affect the quality of life of patients, we must notice that only two studies investigated this particular aspect through established quality-of-life measures. Future studies should include a routine assessment of quality-of-life during the perioperative period and should investigate the correlation between quality-of-life scores and anxiety or other mood measures, in order to achieve a better understanding of the patient’s concerns.

A stream of research is needed to better explore the association of anxiety with characteristics of the tumor, such as location and histology; a fascinating scenario would be also to investigate which of the following has the major impact on patients’ anxiety: the fact of harboring a brain neoplasm or its intrinsic neurophysiological effects.

Considering the assessment methods of anxiety and although HADS was the most used scale, we have to ponder that surgery anxiety is specific and differs from general anxiety [36]; therefore, it needs specific tools designed for surgery patients (which should still be cross validated with established anxiety measures), especially in a brain surgery setting, which is a major surgery with potential cognitive and language impairments. Moreover, there is still no consensus on which assessment tool is best for measuring preoperative anxiety, particularly in brain surgery.

## Strengths and limitations

The main limitation of this review was due to heterogeneity in the included studies: study design, populations, methods, assessment scales, time point of assessment, procedures, lack of postsurgical data to assess anxiety in some studies, data reporting style, and outcomes varied from a study to another not allowing us to perform a quantitative synthesis through meta-analysis. Although we tried to categorize similar studies together, summarizing their findings in order to achieve a more robust level of evidence, sometimes, also qualitative synthesis was difficult due to this heterogeneity and we had to rely on each study individually.

The majority of the included studies dealt with brain tumor patients, who are shown to display great rates of mental distress [18] and greater rates of anxiety and depression if compared to patients with cancer at other sites [56]. The idea of harboring a neoplasm and having surgery in the brain, with the risk of neurological deficits, undoubtedly puts a great burden on the mental health and QOL of patients [25, 46]. This makes our findings not strongly generalizable to all the brain surgical procedures. Despite this, the considerations made in this review may also pertain to non-tumor patients: indeed, even if we included only few studies focused exclusively on non-neoplastic pathology (with epilepsy being the most represented), the rates and complications of preoperative anxiety in this population of neurosurgical patients do not seem to differ consistently from those displayed by patients treated for brain tumors. Future studies are needed in order to establish this evidence and to clarify the phenomenon of preoperative anxiety in the majority of neurosurgical scenarios.

Lack of specific definition in several studies of the type of surgery performed was a limitation of our review, as well as the fact that we decided to include three studies in which the population studied was made of patients undergoing not only brain surgery (at least 40% of the sample) but also spine surgery.

In the majority of the studies, the description of the team setting was not exhaustive, with the professional figure who carried out or supervised the psychological examination of patients not often well characterized; sometimes, it was uncertain if the integrated neurosurgical/psychological team was the standard of care provided every day for the patients or if this was something set up exclusively for the study purposes.

Thus, the evidence we provide should be read with caution; on the other hand, it is, to our knowledge, the first comprehensive effort in brain surgery to elucidate the phenomenon of preoperative anxiety based on the best evidence we can collect today. Given this, it should be considered as giving important clinical clues and as a starting point for future studies in order to provide better care to our patients, evaluating their risk factors, and planning additional treatments for modifiable predictors.

From the methodological side, the strict adherence to PRISMA and Cochrane guidelines and the rigorous assessment of the quality and RoB of the included studies through highly reliable tools can be considered a strength of our work.

## Conclusions

Preoperative anxiety is a common phenomenon that could negatively affect various factors of the perioperative period of brain surgery patients: this is something that should not be neglected to achieve better care through early prevention and optimal management. Patient-centered intervention aiming to prevent or reduce this potential risk factor might be beneficial to the “pre-habilitate” to brain surgery; this needs to be achieved especially through the inclusion of specialized mental health professionals into the standard-of-care team. However, the high heterogeneity and observational nature of the studies highlight the need for further studies in order to corroborate these conclusions.

## Supplementary Information

ESM 1 (PDF 276 kb).

## Data Availability

Not applicable.
