# Supplementary material for: The impact of preoperative anxiety on patients undergoing brain surgery: a systematic review
Source: Neurosurg Rev. 2021 Feb 19;44(6):3047–57. doi: 10.1007/s10143-021-01498-1 (PMC8593022; doi:10.1007/s10143-021-01498-1)
Supplement: Supplementary file 1 — (PDF 276 kb). [file 10143_2021_1498_MOESM1_ESM.pdf]

The impact of preoperative anxiety on patients undergoing brain surgery: a systematic review

Neurosurgical Review

Vittorio Oteri[1*], Anna Martinelli[2], Elisa Crivellaro[3], Francesca Gigli[3]

[1] University of Catania, Catania, Italy

[2] University of Padova, Padova, Italy

[3] University of Milano, Milano, Italy

**Correspondence should be addressed:*

Vittorio Oteri, University of Catania, Department of General Surgery and Medical Specialties, email: oteriv3@gmail.com

Table 1aS. *Risk of bias assessment of the included randomized trials following the RoB 2.0 tool*

Author (Year)	Randomization process	Deviations from intended intervention	Missing outcome data	Measurement of the outcome	Selection of the reported result	Overall
Barrett et al. (1984)	Low	Some concerns	Low	Low	Low	Some concerns
Bekelis et al. (2017)	Low	Low	Some concerns	Low	Low	Some concerns
Kimberger et al. (2007)	Low	Some concerns	Low	Low	Low	Some concerns
Shimony et al. (2016)	Low	Low	Low	Low	Low	Low
Walworth et al. (2008)	Some concerns	Some concerns	Low	Some concerns	Low	Some concerns
Wiles et al. (2017)	Low	Some concerns	Low	Some concerns	Low	Some concerns
Yadav et al. (2017)	Low	Some concerns	Low	Low	Low	Some concerns

Table 1bS. *Risk of bias assessment of the included non-randomized studies following the ROBINS-I tool*

Author (Year)	Confounding	Selection of participants into the study	Classification of interventions	Deviations from intended intervention	Missing outcome data	Measurement of the outcome	Selection of the reported result	Overall
Bunevicius et al. (2013)	Moderate	Low	Low	Low	Low	Low	Low	Moderate
Bunevicius et al. (2014)	Low	Moderate	Low	Low	Moderate	Low	Low	Moderate
Bunevicius et al. (2017)	Low	Low	Low	Low	Low	Low	Low	Low
D'Angelo et al. (2008)	Low	Moderate	Low	Low	Low	Low	Low	Moderate
Goebel et al. (2011)	Moderate	Low	Low	Low	Low	Low	Low	Moderate
Goebel et al. (2013)	Low	Low	Low	Low	Low	Low	Low	Low
Goebel et al. (2013)	Low	Low	Low	Low	Low	Low	Low	Low
Goebel et al. (2018)	Low	Low	Low	Low	Low	Low	Low	Low
Guarnieri et al. (2009)	Moderate	Low	Low	Low	Low	Moderate	Low	Moderate
Hejrati et al. (2019)	Low	Moderate	Low	Low	Moderate	Low	Low	Moderate
Mainio et al. (2003)	Moderate	Moderate	Low	Low	Moderate	Low	Low	Moderate
Palese et al. (2012)	Moderate	Moderate	Low	Low	Low	Low	Low	Moderate
Perks et al. (2009)	Low	Low	Low	Low	Low	Low	Low	Low

Pranckeviciene et al. (2017)	Moderate	Moderate	Low	Low	Moderate	Low	Low	Moderate
Pringle et al. (1999)	Moderate	Low	Low	Low	Moderate	Low	Low	Moderate
Ruis et al. (2017)	Moderate	Low	Low	Low	Low	Low	Low	Moderate
Santini et al. (2012)	Low	Low	Low	Low	Moderate	Low	Low	Moderate
Santini et al. (2012)	Low	Low	Low	Low	Low	Low	Low	Low
Wagner et al. (2019)	Low	Low	Low	Low	Moderate	Low	Low	Moderate
Wrench et al. (2004)	Low	Low	Low	Low	Low	Low	Low	Low

Table 2S. *Quality assessment of the included studies following GRADE approach*

Author (Year)	Study design	Risk of bias	Inconsistency of results	Indirectness of evidence	Imprecision	Publication bias	Large magnitude of effect	Dose-response gradient	Plausible confounding	Overall
Barrett et al. (1984)	Randomized controlled trial	Unclear	Undetected	Undetected	Not serious	Not serious	N/A	N/A	Plausible	High
Bekelis et al. (2017)	Randomized controlled trial	Unclear	Undetected	Undetected	Not serious	Not serious	N/A	N/A	Plausible	High
Bunevicius et al. (2013)	Prospective	Unclear	Undetected	Not serious	Not serious	Not serious	N/A	N/A	Plausible	Low
Bunevicius et al. (2014)	Cross-sectional	Unclear	Undetected	Not serious	Not serious	Not serious	N/A	N/A	Plausible	Low
Bunevicius et al. (2017)	Prospective	Low	Not serious	Not serious	Not serious	Not serious	N/A	N/A	Plausible	Low
D'Angelo et al. (2008)	Prospective	Unclear	Undetected	Not serious	Not serious	Not serious	Large	N/A	Plausible	Moderate
Goebel et al. (2011)	Cross-sectional	Unclear	Not serious	Not serious	Not serious	Not serious	N/A	N/A	Plausible	Low
Goebel et al. (2013)	Cross-sectional	Low	Not serious	Not serious	Not serious	Not serious	N/A	N/A	Plausible	Low
Goebel et al. (2013)	Prospective	Low	Undetected	Undetected	Not serious	Not serious	N/A	N/A	Plausible	Low
Goebel et al. (2018)	Cross-sectional	Low	Undetected	Undetected	Not serious	Not serious	N/A	N/A	Plausible	Low
Guarnieri et al. (2009)	Prospective	Unclear	Not serious	Not serious	Not serious	Not serious	Large	N/A	Plausible	Moderate
Hejrati et al. (2019)	Prospective	Unclear	Undetected	Undetected	Not serious	Not serious	N/A	N/A	Plausible	Low
Kimberger et al. (2007)	Randomized controlled trial	Unclear	Not serious	Undetected	Not serious	Not serious	N/A	N/A	Plausible	High
Mainio et al. (2003)	Prospective	Unclear	Undetected	Undetected	Not serious	Not serious	N/A	N/A	Plausible	Low

Palese et al. (2012)	Cross-sectional	Unclear	Undetected	Undetected	Not serious	Not serious	N/A	N/A	Plausible	Low
Perks et al. (2009)	Cross-sectional	Low	Undetected	Not serious	Not serious	Not serious	N/A	N/A	Plausible	Low
Pranckeviciene et al. (2017)	Cross-sectional	Unclear	Not serious	Undetected	Not serious	Not serious	N/A	N/A	Plausible	Low
Pringle et al. (1999)	Prospective	Unclear	Not serious	Not serious	Not serious	Not serious	N/A	N/A	Plausible	Low
Ruis et al. (2017)	Cross-sectional	Unclear	Undetected	Not serious	Not serious	Not serious	N/A	N/A	Plausible	Low
Santini et al. (2012)	Pilot prospective	Unclear	Undetected	Not serious	Not serious	Not serious	N/A	N/A	Plausible	Low
Santini et al. (2012)	Prospective	Low	Undetected	Not serious	Not serious	Not serious	N/A	N/A	Plausible	Low
Shimony et al. (2016)	Randomized controlled trial	Low	Undetected	Undetected	Not serious	Not serious	N/A	N/A	Plausible	High
Wagner et al. (2019)	Prospective	Unclear	Undetected	Not serious	Not serious	Not serious	N/A	N/A	Plausible	Low
Walworth et al. (2008)	Randomized controlled trial	Unclear	Undetected	Undetected	Not serious	Not serious	N/A	N/A	Plausible	Moderate
Wiles et al. (2017)	Randomized controlled trial	Unclear	Undetected	Undetected	Not serious	Not serious	N/A	N/A	Plausible	High
Wrench et al. (2004)	Prospective	Low	Undetected	Not serious	Not serious	Not serious	N/A	N/A	Plausible	Low
Yadav et al. (2017)	Randomized controlled trial	Unclear	Undetected	Undetected	Not serious	Not serious	N/A	N/A	Plausible	High

N/A – Not applicable.

Table 3S. *Main characteristics of the included studies*

Author (Year)	Type of study	Participants' number	Participants' gender	Participants' mean age in years	Country	Type of operation	Assessment method of anxiety	Time of data collection	Main findings
Studies providing exclusively characteristics of preoperative anxiety and factors correlated									
Bunevicius et al. (2013)	Prospective	90	29% M, 71% F	55.1	Lithuania	Brain tumour surgery (glioma, meningioma, pituitary adenoma, acoustic neuroma)	HADS	Before and after surgery	Preoperative low T3 syndrome not associated with increased risk for preoperative anxiety
Goebel et al. (2013)	Prospective	76<br>• 52 study group<br>• 24 control group	• 33% M, 67% F (study group)<br>• 67% M, 33% F (control group)	• 54 (study group)<br>• 56 (control group)	Germany	Benign intracranial meningioma resection	HADS	1-8 days before surgery (mean 2.4)<br>2-14 days after surgery (mean 6.2)<br>6 months after surgery	20-23% of patients showed high preoperative anxiety. Gender difference not found
Goebel et al. (2018)	Cross-sectional	158	50% M, 50% F	57	Germany	Craniotomy, spinal surgery	APAIS<br>HADS<br>STOA<br>VAS	The day before surgery	APAIS, STOA, VAS can be recommended for assessing brain surgery patients
Mainio et al. (2003)	Prospective	74	41% M, 59% F	48.6	Finland	Intracranial tumour resection	CCEI	1-5 days before surgery<br>3 months and 1 year after surgery	Higher preoperative anxiety in right hemisphere tumour patients.<br>Level of postoperative anxiety declined in right hemisphere tumour patients (significantly if glioma)
Palese et al. (2012)	Cross-sectional	36	72% M, 28% F	46	Italy	Brain tumour surgery	HADS	The day before surgery	Same likelihood of anxiety both in first occurrence and in recurrent neoplasm patients

Pringle et al. (1999)	Prospective	109	57% M, 43% F	17 – 79 (age range)	UK	Intracranial tumour resection	HADS	Before surgery (7 days after radiological diagnosis) After surgery (7 days after initial evaluation)	Higher preoperative anxiety in females with left-sided tumour. Higher preoperative anxiety in patients with glioblastoma.
Preoperative anxiety and preoperative period									
Bunevicius et al. (2014)	Cross-sectional	200	31% M, 69% F	55.8	Lithuania	Brain tumour surgery	HADS	Before surgery	Anxiety impacts on role limitations, pain and general health domains of HRQoL
Goebel et al. (2011)	Cross-sectional	180	48% M, 52% F	52.7	Germany	Brain tumour surgery	APAIS HADS	0-18 days before surgery (mean 2.8)	Ratings of APAIS differed by sex (F more anxious). APAIS proved valid through validation in this specific population
Goebel et al. (2013)	Prospective	172	49% M, 51% F	52	Germany	Brain tumour surgery	APAIS ASDS HADS	1-8 days before surgery (mean 2.4)	Patients with high APAIS score → worse performance in Digit Span Backwards Patients with high ASDS score → worse performance in delayed verbal memory task Patients with high HADS score → worse performance in trail making test
Perks et al. (2009)	Cross-sectional	100	53% M, 47% F	50	USA	Craniotomy, transphenoidal surgery	APAIS VAS	10 days before or the day before surgery	89% had preoperative anxiety, 55% at high levels. Higher incidence in females. Correlation between anxiety and need for information
Pranckeviciene et al. (2017)	Cross-sectional	60	40% M, 60% F	58.3	Lithuania	Brain tumour surgery (meningioma, glioma, acoustic schwannoma, metastatic tumours)	HADS	2-3 days before surgery	Negative impact of distress on preoperative cognitive functioning not confirmed
Preoperative anxiety and postoperative period									
Bunevicius et al. (2017)	Prospective	152	31% M, 69% F	56.9	Lithuania	Brain tumour surgery (glioma, meningioma)	HADS	Before surgery	Preoperative anxiety not associated with shorter survival

D’angelo et al. (2008)	Prospective	72	43% M, 57% F	45.5	Italy	Brain tumour surgery (glioma, meningioma, pituitary adenoma, vestibular schwannoma)	STAI	Before surgery, 1-3-6-12 months after surgery	Correlation between trait preoperative anxiety and depression after surgery
Guarnieri et al. (2009)	Prospective	186	50% M, 50% F	35.8	Brazil	ATL for MTLE-HS	Psychiatric assessment (DSM-IV)	Before surgery	Preoperative anxiety associated with complaints of auras after ATL
Wagner et al. (2019)	Prospective	71	24% M, 76% F	60 (median age)	Germany	Meningioma resection	ASI PTSS STAI	Before surgery, 3 and 12 months after surgery	Preoperative anxiety associated with decrease in QoL during follow-up
Wrench et al. (2008)	Prospective	60<br>• 43 temporal resection<br>• 17 extra-temporal resection	• 53% M, 47% F (temporal resection)<br>• 47% M, 53% F (extra-temporal resection)	• 35 (temporal resection)<br>• 37 (extra-temporal resection)	Austria	Seizure surgery	Austin CEP Interview	Before surgery, at discharge, 1 and 3 months after surgery	History of preoperative anxiety in similar numbers of temporal and extra-temporal patients. Preoperative anxiety levels not differ for left and right-sided resections.
				Awake surgery					
Hejrati et al. (2019)	Prospective	20	55% M, 45% F	56	Switzerland	Awake craniotomy	HADS	Preoperatively on the day of surgery, 3 days and 3 months after surgery	Preoperative anxiety positively correlated with pain in immediate postoperative period
Ruis et al. (2017)	Cross-sectional	70	61% M, 39% F	53	Netherlands	Awake brain tumour surgery	HADS	Within 2 weeks before surgery	25% of patients showed high preoperative anxiety. More anxious: women, younger
Santini et al. (2012)	Prospective	21	48% M, 52% F	25-76 (age range)	Italy	Awake craniotomy	PASS STAI	Before surgery	Correlation between preoperative anxiety and pain during and at the end of operation
Santini et al. (2012)	Prospective	22	45% M, 55% F	91% were < 65 years	Italy	Awake surgery	STAI	Before and after surgery	Anxiety not found to affect cognitive scores
				Interventions to lower Preoperative Anxiety					

Barrett et al. (1984)	Randomized controlled trial	47<br>• 24 oxazepam group<br>• 23 placebo group	• 67% M, 33% F (oxazepam group)<br>• 52% M, 48% F (placebo group)	• 42.5 (oxazepam group)<br>• 50.1 (placebo group)	UK	Craniotomy, laminectomy, peripheral nerve surgery	Degree of anxiety [1-4]	1 h before premedication<br>1 h after premedication	Significant reduction of preoperative anxiety in oxazepam group compared to placebo
Bekelis et al. (2017)	Randomized controlled trial	127<br>• 64 VR group<br>• 63 nonVR group	• 56% M, 44% F (VR group)<br>• 60% M, 40% F (nonVR group)	• 57.3 (VR group)<br>• 53.4 (nonVR group)	USA	Craniotomy, spinal surgery	APAIS<br>VAS	Preoperatively on the day of surgery	Less anxiety in preoperative period in VR group compared to nonVR group
Kimberger et al. (2007)	Randomized controlled trial	80<br>• 20 control group<br>• 20 midazolam group<br>• 20 warming group<br>• 20 combined group	• 65% M, 35% F (control group)<br>• 60% M, 40% F (midazolam group)<br>• 50% M, 50% F (warming group)<br>• 45% M, 55% F (combined group)	• 54.9 (control group)<br>• 50.2 (midazolam group)<br>• 44.5 (warming group)<br>• 46.9 (combined group)	Austria	Neurosurgery	STAI<br>VAS	The day before surgery and before induction of anaesthesia	Pre-operative warming had no influence on preoperative anxiety
Shimony et al. (2016)	Randomized controlled trial	100<br>• 50 placebo group<br>• 50 PGL group	• 55% M, 45% F (placebo group)<br>• 42% M, 58% F (PGL group)	• 51.6 (placebo group)<br>• 52.6 (PGL group)	Israel	Craniotomy	NRS	Before surgery	Pregabalin reduced preoperative anxiety
Walworth et al. (2008)	Randomized controlled trial	27<br>• 14 music intervention group<br>• 13 no music intervention group	44% M, 56% F	46.5	USA	Brain surgery	VAS	Preoperatively on the day of surgery	Positive effect of live music therapy sessions on anxiety and stress
Wiles et al. (2017)	Randomized controlled trial	124<br>• 62 acupuncture group<br>• 62 control group	• 42% M, 58% F (acupuncture group)<br>• 49% M, 51% F (control group)	• 55 (acupuncture group)<br>• 54 (control group)	UK	Neurosurgery	APAIS<br>STAI	Before and after acupuncture in immediate preoperative period	Significant reduction of preoperative anxiety in acupuncture group, with no change in control group
Yadav et al. (2017)	Randomized controlled trial	124<br>• 62 flupirtine group	• 58% M, 42% F (flupirtine group)<br>• 69% M, 31% F (placebo group)	• 40.7 (flupirtine group)<br>• 37.9 (placebo group)	India	Craniotomy	VAS	2 times preoperatively (before preoperative counselling, 2 h	Higher decline of preoperative anxiety in flupirtine treated patients

	• 62 placebo group		after last dose on the evening before surgery)	

→ - correlation; APAIS – Amsterdam Preoperative Anxiety and Information Scale; ASDS – Acute Stress Disorder Scale; ASI –Anxiety Sensitivity Index; ATL – Anterior Temporal Lobectomy; CCEI – Crown-Crisp Experiential Index; CEP – Comprehensive Epilepsy Program; HADS – Hospital Anxiety and Depression Scale; HRQoL – Health Related Quality of Life; MTLE-HS – Mesial Temporal Lobe Epilepsy with Hippocampal Sclerosis; NRS – Numerical Rating Scale [0-10]; PASS – Pain Anxiety Symptoms Scale; PTSS – Post-Traumatic Stress Scale; STAI – Spielberger State Trait Anxiety Inventory; STOA – State Trait Operation Anxiety Inventory; VAS – Visual Analogue Scale; VR – Virtual Reality.
